# Poly[[{μ_3_-2-[4-(2-hy­droxy­eth­yl)piperazin-1-yl]ethane­sulfonato}­silver(I)] trihydrate]

**DOI:** 10.1107/S160053681103008X

**Published:** 2011-08-02

**Authors:** Stephanie M. Bilinovich, Matthew J. Panzner, Wiley J. Youngs, Thomas C. Leeper

**Affiliations:** a University of Akron, Department of Chemistry, Akron, OH 44325-3601, USA

## Abstract

Ethane­sulfonic acid-based buffers like 2-[4-(2-hy­droxy­eth­yl)­piperazin-1-yl]ethane­sulfonic acid (HEPES) are commonly used in biological experiments because of their ability to act as non-coordinating ligands towards metal ions. However, recent work has shown that some of these buffers may in fact coordinate metal ions. The title complex, {[Ag(C_8_H_17_N_2_O_4_S)]·3H_2_O}_*n*_, is a metal–organic framework formed from HEPES and a silver(I) ion. In this polymeric complex, each Ag atom is primarily coordinated by two N atoms in a distorted linear geometry. Weaker secondary bonding inter­actions from the hy­droxy and sulfate O atoms of HEPES complete a distorted seesaw geometry. The crystal structure is stabilized by O—H⋯O hydrogen-bonding interactions.

## Related literature

For other compounds with silver bound to ethane­sulfonic acid derivatives that are used as buffers, see: Jiang, Liu *et al.* (2008 ▶), where HEPES is used, and Jiang, Ma *et al.* (2008 ▶), where MES is used. For background on metal coordination to buffer compounds like HEPES, see: Soares & Conde (2000 ▶); Sokolowska & Bal (2005 ▶). For copper complexes of HEPES inter­fering with protein assays, see: Gregory & Sajdera (1970 ▶); Lleu & Rebel (1991 ▶); Kaushal & Barnes (1986 ▶). For general information on HEPES and related buffers, see: Good *et al.* (1966 ▶). 
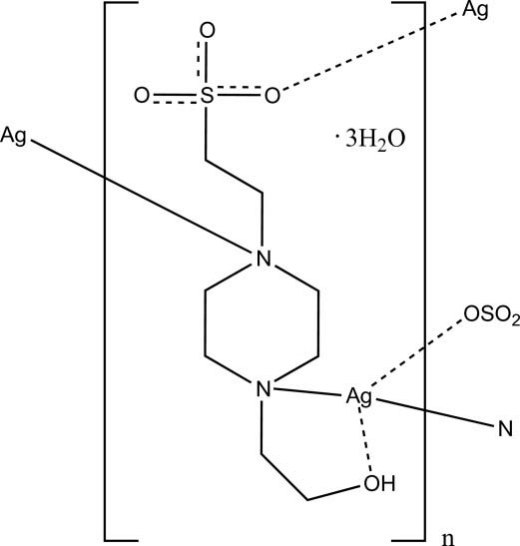


## Experimental

### 

#### Crystal data

[Ag(C_8_H_17_N_2_O_4_S)]·3H_2_O
*M* *_r_* = 399.21
Monoclinic, 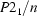

*a* = 11.2811 (19) Å
*b* = 10.0973 (17) Å
*c* = 12.875 (2) Å
β = 90.910 (3)°
*V* = 1466.4 (4) Å^3^
*Z* = 4
Mo *K*α radiation
μ = 1.55 mm^−1^
*T* = 100 K
0.25 × 0.10 × 0.07 mm

#### Data collection

Bruker APEXI CCD diffractometer
Absorption correction: multi-scan (*SADABS*; Bruker, 2001 ▶) *T*_min_ = 0.699, *T*_max_ = 0.900
11308 measured reflections
2946 independent reflections
2446 reflections with *I* > 2σ(*I*)
*R*_int_ = 0.050

#### Refinement

*R*[*F* ^2^ > 2σ(*F* ^2^)] = 0.036
*wR*(*F* ^2^) = 0.090
*S* = 1.09
2946 reflections
190 parameters
9 restraints
H atoms treated by a mixture of independent and constrained refinement
Δρ_max_ = 1.58 e Å^−3^
Δρ_min_ = −0.73 e Å^−3^

### 

Data collection: *SMART* (Bruker, 2007 ▶); cell refinement: *SAINT* (Bruker, 2007 ▶); data reduction: *SAINT*; program(s) used to solve structure: *SHELXS97* (Sheldrick, 2008 ▶); program(s) used to refine structure: *SHELXL97* (Sheldrick, 2008 ▶); molecular graphics: *Mercury* (Macrae *et al.*, 2006 ▶); software used to prepare material for publication: *SHELXTL* (Sheldrick, 2008 ▶).

## Supplementary Material

Crystal structure: contains datablock(s) I, global. DOI: 10.1107/S160053681103008X/mw2014sup1.cif

Structure factors: contains datablock(s) I. DOI: 10.1107/S160053681103008X/mw2014Isup2.hkl

Additional supplementary materials:  crystallographic information; 3D view; checkCIF report

## Figures and Tables

**Table:** 

Ag1—N1	2.266 (3)
Ag1—N2	2.280 (3)
Ag1—O2^i^	2.666 (2)
Ag1—O4	2.581 (2)

**Table:** 

N1—Ag1—N2	167.73 (11)
N1—Ag1—O2^i^	92.58 (8)
N1—Ag1—O4	115.41 (8)
N2^ii^—Ag1—O2^i^	94.22 (9)
N2—Ag1—O4	75.16 (9)
O2^i^—Ag1—O4^ii^	87.18 (7)

Symmetry codes: (i) 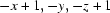; (ii) 
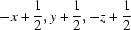
.

**Table 2:** Hydrogen-bond geometry (Å, °)

*D*—H⋯*A*	*D*—H	H⋯*A*	*D*⋯*A*	*D*—H⋯*A*
O7—H7*C*⋯O5^iii^	0.85 (2)	1.96 (2)	2.777 (4)	161 (4)
O6—H6*A*⋯O3^iv^	0.84 (2)	1.88 (2)	2.706 (4)	166 (4)
O6—H6*C*⋯O1^v^	0.86 (2)	2.02 (2)	2.868 (4)	169 (4)
O5—H5*C*⋯O7^vi^	0.85 (2)	2.01 (2)	2.834 (4)	163 (5)
O5—H5*D*⋯O6^vii^	0.86 (2)	2.02 (2)	2.864 (4)	171 (4)
O4—H4⋯O6^vii^	0.84	1.89	2.726 (4)	178

Symmetry codes: (iii) 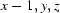; (iv) 
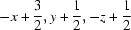
; (v) 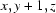; (vi) 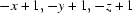; (vii) 
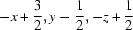
.

**Fig. 1.**
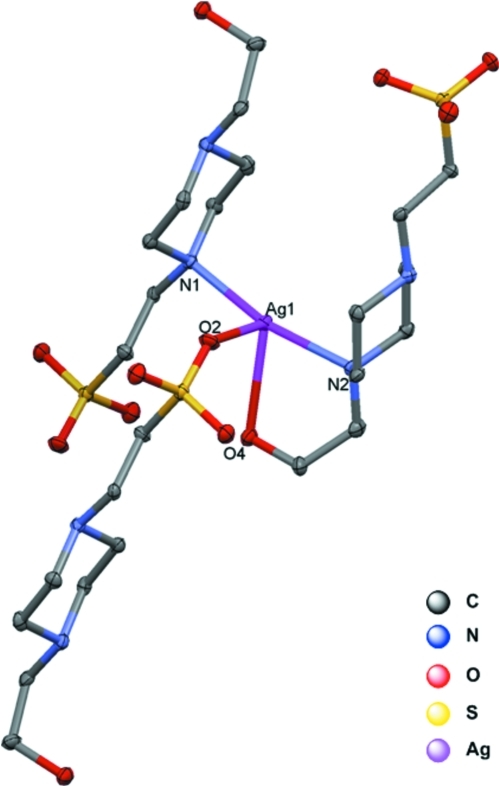
Thermal ellipsoid plot depicting the bonding interaction of three HEPES molecules with one silver(I) atom affording a see-saw geometry. Hydrogen atoms and additional symmetry related molecules removed for clarity. Displacement ellipsoids shown at the 50% probability level.

**Fig. 2.**
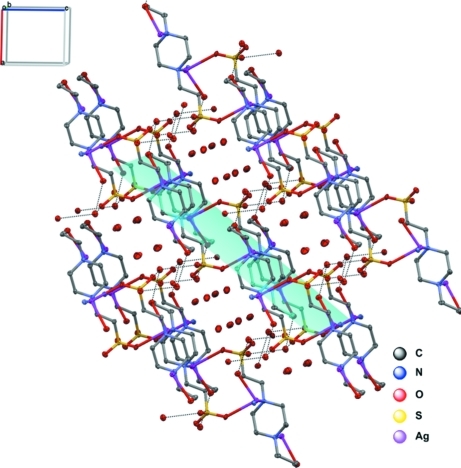
Packing view down the b axis of the title compound depicting the three-dimensional network created by the specific hydrogen bonding interaction of water molecule O6 with layers of Ag(I)-HEPES.

## supplementary crystallographic information

### Comment

HEPES is one of twelve buffers introduced by Good and coworkers as ideal for biological studies based on their physiologically relevant buffering capacities. It was also initially stated that HEPES would not form complexes with metals (Good *et al.* 1966). However, several studies show that HEPES can form complexes with copper that account for interferences in protein quantification assays like Lowry and BCA (Gregory & Sajdera, 1970; Lleu & Rebel, 1991; Kaushal & Barnes, 1986). In addition, recent electrochemical and spectroscopic studies have shown that HEPES can act as a weak chelator with lead(II) and copper(II) (Soares & Conde, 2000; Sokolowska & Bal, 2005). Due to the recent interest in studying the role that silver(I)-containing compounds play as medicinal agents, the identification of buffers that prevent precipitation or complex formation with silver(I) ion are needed. Based on their established properties it was surmised that one of Good's non-coordinating buffers would be ideal for such investigations. However, as is evident from the title compound, HEPES does in fact form a stable complex with silver(I) ion making it a poor choice for use with systems containing silver ions. In the title compound, the Ag(I) ion is coordinated by one nitrogen atom and one hydroxyl oxygen atom of a HEPES molecule, one nitrogen atom of a second HEPES molecule, and one sulfate oxygen atom from a third HEPES molecule affording a distorted see-saw geometry about the metal center. Precedence for similar weak Ag···O interactions as well as the distorted see-saw geometry can be found in the literature and by a search of the Cambridge Crystallographic Database (Jiang, Liu *et al.* 2008). As is indicated by the bond distances, the nitrogen atoms form covalent bonds with the Ag(I) atom (2.266 (3) and 2.280 (3) Å) in a near linear fashion (N—Ag—N = 167.73 (11)°). The interactions of the hydroxyl and sulfate oxygen atoms with the Ag(I) ion are weaker (HO···Ag = 2.581 (2) and O_2_SO···Ag = 2.666 (2) Å) but well within the sum of the Van der Waals radii  for silver and oxygen (3.24 Å). The interaction of  HEPES with Ag(I) affords a layered two-dimensional network perpendicular to the c axis, and these layers are further associated into a three-dimensional network through hydrogen bonding with the water molecules, directly via water O6, of the structure (Figure 2).

### Experimental

A 250 ml 1 *M* stock solution of HEPES (4-(2-hydroxyethyl)-1-piperazineethanesulfonic acid) buffer was prepared by dissolving 59.57 grams of HEPES in 200 ml of water and adjusting the pH to 7 with 5*M* NaOH before adjusting the volume to 250 ml. A 250 ml stock solution of 1*M* silver nitrate was prepared by dissolving 41.96 grams in 250 ml of water. To form the compound, 90 ml of the 1*M* silver nitrate stock solution was added to 10 ml of the 1*M* HEPES buffer stock solution to yield final concentrations of 0.9*M* silver nitrate and 0.1*M* HEPES in the solution. After one hour the experiment had gone to completion and long gray needle-like crystals were observed.

### Refinement

Methylene H atoms were calculated with a C—H distances of 0.99Å and constrained to ride on the parent atom with *U*_iso_(H) = 1.2 *U*_eq_(C). The hydroxyl H atom of the HEPES molecule and the H atoms of the solvent water molecules were found in the difference Fourier map. The first was included as a riding contribution with an O—H distance of 0.84 Å and *U*_iso_(H) = 1.5 *U*_eq_(O) while the others were refined with fixed displacement parameters (*U*_iso_(H) = 1.5 *U*_eq_(O)).

### Crystal data

**Table:**

[Ag(C_8_H_17_N_2_O_4_S)]·3H_2_O	*F*(000) = 816
*M**_r_* = 399.21	*D*_x_ = 1.808 Mg m^−^^3^
Monoclinic, *P*2_1_/*n*	Mo *K*α radiation, λ = 0.71073 Å
Hall symbol: -P 2yn	Cell parameters from 1601 reflections
*a* = 11.2811 (19) Å	θ = 2.7–21.3°
*b* = 10.0973 (17) Å	µ = 1.55 mm^−^^1^
*c* = 12.875 (2) Å	*T* = 100 K
β = 90.910 (3)°	Column, colorless
*V* = 1466.4 (4) Å^3^	0.25 × 0.10 × 0.07 mm
*Z* = 4	

### Data collection

**Table:**

Bruker APEXI CCD diffractometer	2946 independent reflections
Radiation source: fine-focus sealed tube	2446 reflections with *I* > 2σ(*I*)
graphite	*R*_int_ = 0.050
φ and ω scans	θ_max_ = 26.3°, θ_min_ = 2.4°
Absorption correction: multi-scan (*SADABS*; Bruker 2001)	*h* = −14→14
*T*_min_ = 0.699, *T*_max_ = 0.900	*k* = −12→11
11308 measured reflections	*l* = −16→16

### Refinement

**Table:**

Refinement on *F*^2^	Primary atom site location: structure-invariant direct methods
Least-squares matrix: full	Secondary atom site location: difference Fourier map
*R*[*F*^2^ > 2σ(*F*^2^)] = 0.036	Hydrogen site location: inferred from neighbouring sites
*wR*(*F*^2^) = 0.090	H atoms treated by a mixture of independent and constrained refinement
*S* = 1.09	*w* = 1/[σ^2^(*F*_o_^2^) + (0.0356*P*)^2^] where *P* = (*F*_o_^2^ + 2*F*_c_^2^)/3
2946 reflections	(Δ/σ)_max_ = 0.001
190 parameters	Δρ_max_ = 1.58 e Å^−^^3^
9 restraints	Δρ_min_ = −0.73 e Å^−^^3^

### Special details

**Table:**

Experimental. Two A level alerts are generated by cif check: Angle Calc 87.45 (5), Rep 94.22 (9), Dev..135.40 Sigma N2-AG1-O2 Angle Calc 89.17 (5), Rep 87.18 (7), Dev..135.40 Sigma O2-AG1-O4 Both of the reported angles were verified during refinement with SHELXL-97 and can be confirmed by analyzing the resulting cif with Mercury.
Geometry. All e.s.d.'s (except the e.s.d. in the dihedral angle between two l.s. planes) are estimated using the full covariance matrix. The cell e.s.d.'s are taken into account individually in the estimation of e.s.d.'s in distances, angles and torsion angles; correlations between e.s.d.'s in cell parameters are only used when they are defined by crystal symmetry. An approximate (isotropic) treatment of cell e.s.d.'s is used for estimating e.s.d.'s involving l.s. planes.
Refinement. Refinement of *F*^2^ against ALL reflections. The weighted *R*-factor *wR* and goodness of fit *S* are based on *F*^2^, conventional *R*-factors *R* are based on *F*, with *F* set to zero for negative *F*^2^. The threshold expression of *F*^2^ > σ(*F*^2^) is used only for calculating *R*-factors(gt) *etc*. and is not relevant to the choice of reflections for refinement. *R*-factors based on *F*^2^ are statistically about twice as large as those based on *F*, and *R*- factors based on ALL data will be even larger. Distance and angle restraints were applied to the hydrogen atoms associated with the three solvent water molecules found from the difference Fourier map.

### Fractional atomic coordinates and isotropic or equivalent isotropic displacement parameters (Å^2^)

**Table:**

	*x*	*y*	*z*	*U*_iso_*/*U*_eq_	
Ag1	0.38547 (2)	0.14705 (2)	0.29401 (2)	0.01471 (11)	
S1	0.68653 (8)	−0.16901 (8)	0.41427 (7)	0.0135 (2)	
O1	0.6794 (2)	−0.3109 (2)	0.39089 (19)	0.0177 (6)	
O2	0.7190 (2)	−0.1418 (2)	0.52171 (19)	0.0189 (6)	
O3	0.7607 (2)	−0.1011 (3)	0.3391 (2)	0.0196 (6)	
O4	0.5744 (2)	0.2274 (2)	0.39058 (19)	0.0175 (6)	
H4	0.6437	0.2101	0.3717	0.026*	
O5	0.8863 (3)	0.3405 (3)	0.4864 (2)	0.0317 (7)	
H5C	0.883 (5)	0.412 (3)	0.452 (3)	0.048*	
H5D	0.862 (4)	0.281 (3)	0.444 (3)	0.048*	
O6	0.7024 (3)	0.6635 (2)	0.1703 (2)	0.0211 (6)	
H6A	0.707 (4)	0.582 (2)	0.158 (3)	0.032*	
H6C	0.704 (4)	0.666 (4)	0.2369 (15)	0.032*	
O7	0.0771 (3)	0.4128 (3)	0.6131 (2)	0.0321 (7)	
H7C	0.027 (3)	0.374 (4)	0.574 (3)	0.048*	
H7D	0.143 (2)	0.375 (4)	0.617 (4)	0.048*	
N1	0.3695 (2)	−0.0753 (3)	0.2742 (2)	0.0123 (6)	
N2	0.3799 (3)	0.3721 (3)	0.2809 (2)	0.0142 (7)	
C1	0.2842 (3)	−0.1321 (3)	0.3490 (3)	0.0139 (7)	
H1B	0.3071	−0.1046	0.4203	0.017*	
H1A	0.2879	−0.2300	0.3458	0.017*	
C2	0.3296 (3)	−0.1120 (4)	0.1675 (3)	0.0148 (8)	
H2B	0.3345	−0.2093	0.1593	0.018*	
H2A	0.3832	−0.0711	0.1166	0.018*	
C3	0.2954 (3)	0.4323 (3)	0.3552 (3)	0.0153 (8)	
H3B	0.2998	0.5300	0.3503	0.018*	
H3A	0.3182	0.4064	0.4269	0.018*	
C4	0.3408 (3)	0.4125 (3)	0.1743 (3)	0.0140 (7)	
H4A	0.3948	0.3735	0.1228	0.017*	
H4B	0.3454	0.5101	0.1681	0.017*	
C5	0.4886 (3)	−0.1349 (3)	0.2912 (3)	0.0124 (7)	
H5B	0.5429	−0.1006	0.2379	0.015*	
H5A	0.4826	−0.2321	0.2820	0.015*	
C6	0.5408 (3)	−0.1060 (4)	0.3980 (3)	0.0144 (8)	
H6D	0.4893	−0.1455	0.4512	0.017*	
H6B	0.5421	−0.0090	0.4091	0.017*	
C7	0.5006 (3)	0.4236 (3)	0.3009 (3)	0.0164 (8)	
H7B	0.4966	0.5212	0.3076	0.020*	
H7A	0.5504	0.4031	0.2404	0.020*	
C8	0.5599 (3)	0.3672 (3)	0.3982 (3)	0.0187 (8)	
H8B	0.6384	0.4094	0.4086	0.022*	
H8A	0.5111	0.3881	0.4593	0.022*	

### Atomic displacement parameters (Å^2^)

**Table:**

	*U*^11^	*U*^22^	*U*^33^	*U*^12^	*U*^13^	*U*^23^
Ag1	0.01836 (18)	0.00864 (16)	0.01706 (17)	−0.00008 (10)	−0.00190 (12)	−0.00012 (11)
S1	0.0165 (5)	0.0111 (4)	0.0130 (4)	0.0014 (3)	−0.0011 (4)	0.0000 (3)
O1	0.0216 (14)	0.0125 (12)	0.0190 (14)	0.0033 (10)	−0.0010 (12)	−0.0018 (11)
O2	0.0219 (15)	0.0213 (14)	0.0133 (13)	0.0004 (11)	−0.0053 (11)	−0.0043 (11)
O3	0.0214 (15)	0.0181 (13)	0.0194 (14)	−0.0005 (11)	0.0043 (12)	0.0007 (11)
O4	0.0177 (13)	0.0118 (13)	0.0228 (14)	0.0004 (10)	−0.0014 (11)	0.0000 (11)
O5	0.0340 (18)	0.0341 (18)	0.0270 (17)	−0.0089 (14)	−0.0016 (15)	−0.0027 (13)
O6	0.0262 (16)	0.0168 (14)	0.0203 (14)	0.0012 (11)	0.0016 (13)	−0.0001 (12)
O7	0.0300 (18)	0.0325 (18)	0.0334 (18)	0.0154 (14)	−0.0098 (14)	−0.0091 (14)
N1	0.0110 (16)	0.0115 (15)	0.0142 (16)	−0.0011 (11)	0.0006 (12)	0.0015 (12)
N2	0.0151 (17)	0.0123 (15)	0.0151 (16)	−0.0013 (11)	0.0008 (13)	0.0031 (12)
C1	0.0163 (19)	0.0119 (18)	0.0134 (18)	−0.0016 (14)	0.0006 (15)	−0.0001 (14)
C2	0.019 (2)	0.0154 (18)	0.0097 (17)	−0.0001 (14)	0.0015 (15)	−0.0012 (14)
C3	0.0180 (19)	0.0124 (18)	0.0154 (19)	0.0057 (14)	0.0003 (15)	−0.0006 (15)
C4	0.0175 (19)	0.0110 (18)	0.0135 (18)	0.0013 (14)	0.0036 (15)	0.0020 (14)
C5	0.0164 (19)	0.0091 (17)	0.0117 (17)	0.0020 (13)	0.0000 (15)	0.0015 (14)
C6	0.0147 (19)	0.0160 (18)	0.0125 (18)	0.0021 (14)	−0.0026 (15)	−0.0012 (15)
C7	0.0143 (19)	0.0111 (18)	0.024 (2)	−0.0033 (14)	0.0002 (16)	0.0017 (16)
C8	0.019 (2)	0.0133 (19)	0.024 (2)	−0.0012 (14)	−0.0048 (17)	−0.0046 (16)

### Geometric parameters (Å, °)

**Table:**

Ag1—N1	2.266 (3)	C1—C4^ii^	1.506 (5)
Ag1—N2	2.280 (3)	C1—H1B	0.9900
Ag1—O2^i^	2.666 (2)	C1—H1A	0.9900
Ag1—O4	2.581 (2)	C2—C3^ii^	1.503 (5)
S1—O2	1.452 (3)	C2—H2B	0.9900
S1—O3	1.461 (3)	C2—H2A	0.9900
S1—O1	1.466 (3)	C3—C2^iii^	1.503 (5)
S1—C6	1.772 (4)	C3—H3B	0.9900
O4—C8	1.425 (4)	C3—H3A	0.9900
O4—H4	0.8400	C4—C1^iii^	1.506 (5)
O5—H5C	0.851 (19)	C4—H4A	0.9900
O5—H5D	0.855 (18)	C4—H4B	0.9900
O6—H6A	0.839 (18)	C5—C6	1.515 (5)
O6—H6C	0.858 (18)	C5—H5B	0.9900
O7—H7C	0.848 (19)	C5—H5A	0.9900
O7—H7D	0.838 (19)	C6—H6D	0.9900
N1—C5	1.485 (4)	C6—H6B	0.9900
N1—C2	1.486 (4)	C7—C8	1.521 (5)
N1—C1	1.486 (4)	C7—H7B	0.9900
N2—C7	1.477 (4)	C7—H7A	0.9900
N2—C3	1.490 (4)	C8—H8B	0.9900
N2—C4	1.492 (4)	C8—H8A	0.9900
			
N1—Ag1—N2	167.73 (11)	C3^ii^—C2—H2A	109.2
N1—Ag1—O2^i^	92.58 (8)	H2B—C2—H2A	107.9
N1—Ag1—O4	115.41 (8)	N2—C3—C2^iii^	111.2 (3)
N2^iii^—Ag1—O2^i^	94.22 (9)	N2—C3—H3B	109.4
N2—Ag1—O4	75.16 (9)	C2^iii^—C3—H3B	109.4
O2^i^—Ag1—O4^iii^	87.18 (7)	N2—C3—H3A	109.4
O2—S1—O3	113.85 (16)	C2^iii^—C3—H3A	109.4
O2—S1—O1	113.13 (14)	H3B—C3—H3A	108.0
O3—S1—O1	110.65 (15)	N2—C4—C1^iii^	111.3 (3)
O2—S1—C6	105.35 (16)	N2—C4—H4A	109.4
O3—S1—C6	107.02 (16)	C1^iii^—C4—H4A	109.4
O1—S1—C6	106.21 (16)	N2—C4—H4B	109.4
C8—O4—H4	109.5	C1^iii^—C4—H4B	109.4
H5C—O5—H5D	105 (4)	H4A—C4—H4B	108.0
H6A—O6—H6C	103 (3)	N1—C5—C6	113.1 (3)
H7C—O7—H7D	113 (4)	N1—C5—H5B	109.0
C5—N1—C2	107.1 (3)	C6—C5—H5B	109.0
C5—N1—C1	109.9 (3)	N1—C5—H5A	109.0
C2—N1—C1	108.2 (3)	C6—C5—H5A	109.0
C5—N1—Ag1	108.4 (2)	H5B—C5—H5A	107.8
C2—N1—Ag1	111.9 (2)	C5—C6—S1	112.6 (2)
C1—N1—Ag1	111.2 (2)	C5—C6—H6D	109.1
C7—N2—C3	110.0 (3)	S1—C6—H6D	109.1
C7—N2—C4	108.8 (3)	C5—C6—H6B	109.1
C3—N2—C4	107.2 (3)	S1—C6—H6B	109.1
C7—N2—Ag1	108.3 (2)	H6D—C6—H6B	107.8
C3—N2—Ag1	112.1 (2)	N2—C7—C8	113.8 (3)
C4—N2—Ag1	110.4 (2)	N2—C7—H7B	108.8
N1—C1—C4^ii^	111.7 (3)	C8—C7—H7B	108.8
N1—C1—H1B	109.3	N2—C7—H7A	108.8
C4^ii^—C1—H1B	109.3	C8—C7—H7A	108.8
N1—C1—H1A	109.3	H7B—C7—H7A	107.7
C4^ii^—C1—H1A	109.3	O4—C8—C7	111.4 (3)
H1B—C1—H1A	108.0	O4—C8—H8B	109.4
N1—C2—C3^ii^	112.0 (3)	C7—C8—H8B	109.4
N1—C2—H2B	109.2	O4—C8—H8A	109.4
C3^ii^—C2—H2B	109.2	C7—C8—H8A	109.4
N1—C2—H2A	109.2	H8B—C8—H8A	108.0
			
N2—Ag1—N1—C5	130.6 (5)	C7—N2—C4—C1^iii^	−177.5 (3)
N2—Ag1—N1—C2	12.7 (6)	C3—N2—C4—C1^iii^	−58.5 (4)
N2—Ag1—N1—C1	−108.5 (5)	Ag1—N2—C4—C1^iii^	63.8 (3)
N1—Ag1—N2—C7	−131.4 (5)	C2—N1—C5—C6	−179.8 (3)
N1—Ag1—N2—C3	107.1 (5)	C1—N1—C5—C6	−62.4 (4)
N1—Ag1—N2—C4	−12.4 (6)	Ag1—N1—C5—C6	59.3 (3)
C5—N1—C1—C4^ii^	−172.7 (3)	N1—C5—C6—S1	−176.3 (2)
C2—N1—C1—C4^ii^	−56.0 (4)	O2—S1—C6—C5	−175.8 (2)
Ag1—N1—C1—C4^ii^	67.3 (3)	O3—S1—C6—C5	62.7 (3)
C5—N1—C2—C3^ii^	174.6 (3)	O1—S1—C6—C5	−55.5 (3)
C1—N1—C2—C3^ii^	56.1 (4)	C3—N2—C7—C8	74.0 (4)
Ag1—N1—C2—C3^ii^	−66.7 (3)	C4—N2—C7—C8	−168.8 (3)
C7—N2—C3—C2^iii^	176.5 (3)	Ag1—N2—C7—C8	−48.8 (3)
C4—N2—C3—C2^iii^	58.4 (4)	N2—C7—C8—O4	61.9 (4)
Ag1—N2—C3—C2^iii^	−62.9 (3)		

Symmetry codes: (i) −*x*+1, −*y*, −*z*+1; (ii) −*x*+1/2, *y*−1/2, −*z*+1/2; (iii) −*x*+1/2, *y*+1/2, −*z*+1/2.

### Hydrogen-bond geometry (Å, °)

**Table:**

*D*—H···*A*	*D*—H	H···*A*	*D*···*A*	*D*—H···*A*
O7—H7C···O5^iv^	0.85 (2)	1.96 (2)	2.777 (4)	161 (4)
O6—H6A···O3^v^	0.84 (2)	1.88 (2)	2.706 (4)	166 (4)
O6—H6C···O1^vi^	0.86 (2)	2.02 (2)	2.868 (4)	169 (4)
O5—H5C···O7^vii^	0.85 (2)	2.01 (2)	2.834 (4)	163 (5)
O5—H5D···O6^viii^	0.86 (2)	2.02 (2)	2.864 (4)	171 (4)
O4—H4···O6^viii^	0.84	1.89	2.726 (4)	178

Symmetry codes: (iv) *x*−1, *y*, *z*; (v) −*x*+3/2, *y*+1/2, −*z*+1/2; (vi) *x*, *y*+1, *z*; (vii) −*x*+1, −*y*+1, −*z*+1; (viii) −*x*+3/2, *y*−1/2, −*z*+1/2.
